# Psychosocial Barriers and Social Perceptions in Oncology Patients with Tracheostomy: Case–Control Study

**DOI:** 10.3390/curroncol33010022

**Published:** 2025-12-31

**Authors:** Tomasz Jurys, Milena Orzażewska, Karolina Klimek, Eliza Działach, Jarosław Markowski, Mateusz Grajek

**Affiliations:** 1 Department of Rehabilitation, Faculty of Health Sciences in Katowice, Medical University of Silesia in Katowice, 40751 Katowice, Poland; 2 Department of Public Health, Faculty of Public Health in Bytom, Medical University of Silesia in Katowice, 41902 Bytom, Poland; s85066@365.sum.edu.pl (M.O.); edzialach@sum.edu.pl (E.D.); mgrajek@sum.edu.pl (M.G.); 3 Department of Health Economics and Health Management, School of Health Sciences in Bytom, Medical University of Silesia in Katowice, 41902 Bytom, Poland; ksobczyk@sum.edu.pl; 4 Department of Otorhinolarygology, Faculty of Medicine in Katowice, Medical University of Silesia in Katowice, 40027 Katowice, Poland; jmarkowski@sum.edu.pl

**Keywords:** tracheostomy, head and neck cancer, psychosocial barriers, social acceptance, stigma and quality of life

## Abstract

Patients with respiratory tract malignancies who undergo tracheostomy often experience profound psychosocial challenges related to visible anatomical changes and altered communication. The aim of this study was to evaluate psychosocial barriers and perceived social acceptance in patients living with a tracheostomy, compared with patients treated for similar cancers without requiring a tracheostomy. A matched case–control study with frequency matching at the group level was conducted including 150 patients with permanent tracheostomies and 150 matched controls treated with organ-preserving approaches. Groups were frequency-matched at the group level based on age, sex, primary tumor site, and disease stage at diagnosis. Participants completed a study-specific questionnaire assessing social withdrawal, self-consciousness, and perceived reactions of others using a five-point Likert scale. A composite Psychosocial Barrier Score was calculated, and subgroup analyses examined differences according to gender and age. Patients with tracheostomies demonstrated significantly higher psychosocial burden than controls, with markedly elevated composite scores and higher endorsement of stigma-related items. Female and younger patients within the tracheostomy group reported the greatest psychosocial difficulties, including increased social avoidance and reduced confidence in public settings. In contrast, gender- and age-related differences were minimal in the control group. These findings indicate that tracheostomy is strongly associated with heightened psychosocial barriers and perceived social stigma, particularly among younger and female patients. Integrating targeted psychosocial support into routine post-treatment care may be essential to improve social reintegration and quality of life in this population.

## Simple Summary

Patients with cancers of the throat or windpipe sometimes need a surgical opening in the neck called a tracheostomy to help them breathe. Although this procedure can save lives, it also changes how a person looks and speaks, which may affect everyday social interactions. In this study, we compared people living with a tracheostomy to people with similar cancers who did not need one. We found that those with a tracheostomy felt more judged by others, avoided social situations more often, and reported lower confidence in public. Women and younger adults were especially affected. These results show that the emotional and social consequences of a tracheostomy can be significant and should not be overlooked. The findings highlight the need for better support programs, counseling, and rehabilitation services to help patients adjust and feel more comfortable returning to their normal lives.

## 1. Introduction

Head and neck malignancies of the respiratory tract (particularly laryngeal and tracheal carcinomas) frequently necessitate aggressive therapeutic approaches that can profoundly affect essential physiological functions such as breathing, phonation, and airway protection [1,2]. In advanced stages of disease or in the presence of critical airway compromise, a surgical tracheotomy may be required, resulting in a tracheostomy that provides an alternative and secure airway. Although often life-saving, the creation of a permanent tracheostoma entails substantial anatomical and functional consequences, including visible alterations to the neck and, in many cases, significant changes in vocal communication [3,4].

Beyond the immediate clinical implications, patients living with a tracheostomy as a consequence of cancer treatment face considerable long-term psychosocial challenges [5,6]. The stoma serves as a persistent and highly visible reminder of illness, which may influence self-perception as well as interactions with others. Altered speech, ranging from dysphonia to aphonia, combined with the conspicuous appearance of the tracheostomy tube, can draw unsolicited attention and may contribute to embarrassment, diminished self-esteem, and heightened anxiety in social environments [6,7,8]. While individuals with comparable malignancies treated through organ-preserving modalities are not exempt from psychosocial burden, they frequently avoid the specific visibility-related stressors associated with tracheostomy [7,9].

Clinical observations and emerging empirical evidence indicate that patients with tracheostomies may experience various forms of social stigmatization or reduced social acceptance [7,10]. Many patients report sensing differential treatment from people without health limitations (“healthy others”), often attributing these responses to their altered voice or the presence of the tracheostoma [8]. These psychosocial barriers can manifest as fear of being stared at or judged, reluctance to participate in social or occupational activities, and progressive withdrawal from interpersonal relationships and public life.

Demographic factors, particularly gender and age, have been suggested as potential moderators of psychosocial vulnerability in this population [11,12,13]. Preliminary reports and smaller-scale studies propose that women may experience elevated distress associated with visible bodily changes, possibly due to societal expectations regarding appearance and gendered patterns of social evaluation. Likewise, younger patients may encounter greater psychosocial disruption, as alterations in appearance and communication can interfere with work, family roles, and broader social engagement at a life stage characterized by high social activity and professional aspirations. Despite these indications, rigorous comparative research quantifying the magnitude of psychosocial barriers among tracheostomized versus non-tracheostomized cancer patients remains limited [14,15,16].

We hypothesized that patients living with a permanent tracheostomy would exhibit significantly higher psychosocial barrier scores than patients treated for comparable respiratory tract malignancies without tracheostomy. Accordingly, the objective of this study was to evaluate psychosocial barriers in patients living with a tracheostomy in comparison with matched patients treated without tracheostomy, with particular emphasis on perceived social acceptance in everyday interactions.

## 2. Materials and Methods

A matched case–control study with frequency matching at the group level was conducted at a tertiary oncology center to compare psychosocial outcomes between patients with respiratory tract malignancies living with a tracheostomy and patients treated for similar cancers without requiring a tracheostomy. Eligible participants were adult patients with respiratory tract malignancies who had completed primary oncologic treatment. Patients with severe uncontrolled psychiatric disorders or inability to complete the questionnaire were excluded. The clinical (tracheostomy) group consisted of 150 patients diagnosed predominantly with upper respiratory tract malignancies, most commonly laryngeal cancer, with additional cases of tracheal and subglottic tumors. All patients in this group had undergone surgical tracheotomy resulting in a permanent tracheostoma and had completed primary cancer treatment, including surgery and, when indicated, adjuvant radiotherapy or chemoradiotherapy. At the time of data collection, all were actively living with a tracheostomy. The control group included 150 patients with comparable diagnoses and disease stages who were managed without the need for a tracheostomy. These patients were treated with organ-preserving approaches, such as partial laryngectomy or definitive chemoradiotherapy, or underwent surgical management of early-stage disease that did not require the creation of a permanent stoma. To minimize confounding, frequency matching at the group level was performed based on key clinical variables, including primary tumor site and disease stage at diagnosis. These variables were selected due to their direct relevance to treatment intensity, functional impairment, and psychosocial outcomes. The tracheostomy and control groups were aligned with respect to age distribution, sex, primary tumor site, and disease stage. Matching was conducted at the group level rather than through individual pairwise matching. No propensity score methods were applied, as the primary aim of the study was descriptive comparison rather than causal inference.

All participants had completed primary oncologic treatment prior to enrollment. Time since treatment completion was recorded and did not differ significantly between groups. Although age and sex were included as frequency-matching variables, exploratory subgroup analyses stratified by age and gender were conducted within each group to examine potential heterogeneity in psychosocial burden. These analyses were descriptive in nature. Patients with clinically evident severe psychiatric disorders requiring acute psychiatric treatment were excluded. Formal psychiatric screening instruments (e.g., HADS, PHQ-9, GAD-7) were not systematically administered; psychiatric status was assessed based on medical records and clinical judgment during routine follow-up. Psychosocial assessments were performed at a single post-treatment time point. Of the 327 patients who met eligibility criteria, 300 consented to participate and were included in the final analysis, while 27 declined participation. The recruitment period for study participants covered the period from January 2025 to July 2025. The study was exploratory and that no a priori sample size calculation was performed.

### 2.1. Ethics

The study was conducted in accordance with the ethical principles outlined in the Declaration of Helsinki. The research protocol received approval from the Institutional Review Board of the Medical University of Silesia in Katowice, Poland (approval number: PCN/CBN/0052/KB/302/22/23). All participants were fully informed about the purpose and procedures of the study and provided written informed consent prior to enrollment. Patients were recruited consecutively during routine follow-up visits in the outpatient oncology clinic. Following confirmation of eligibility and acquisition of consent, baseline demographic characteristics (including age, sex, and educational level) and clinical data (tumor type, stage at diagnosis, and time elapsed since completion of treatment) were systematically collected.

### 2.2. Assessment Tool Development

To evaluate psychosocial barriers and perceived social acceptance, a structured questionnaire was developed specifically for this study. The instrument was informed by a review of the relevant literature and preliminary semi-structured interviews with patients, ensuring alignment with domains most commonly affected in individuals with head and neck malignancies. The questionnaire comprised a series of statements assessing patients’ emotions, behaviors, and interpersonal experiences following completion of cancer treatment. Participants rated their agreement with each statement using a five-point Likert scale (1 = strongly disagree, 5 = strongly agree). Dichotomization of Likert-scale responses was applied for descriptive clarity and ease of interpretation. Inferential analyses focused primarily on composite scale scores, and results should be interpreted accordingly. Items were constructed to capture core psychosocial domains, including social withdrawal, self-consciousness related to the tracheostomy, and perceived reactions of others. The following statements represent the key items included in the measure:

- “I avoid social gatherings because of my medical condition.”
- “I feel that people stare at me or my tracheostomy tube in public.”
- “I worry that healthy people find it uncomfortable to communicate with me because of my tracheostomy.”
- “I feel less confident in social situations now than I did before my illness.”
- “I believe that some people avoid me due to the changes in my appearance or voice.”

The development of the questionnaire followed a multi-step process. Initially, a focused narrative review of the literature on psychosocial functioning, stigma, and quality of life in patients with head and neck cancer and tracheostomy was conducted to identify key domains relevant to social acceptance and interpersonal functioning. These domains included social withdrawal, perceived public reactions, self-consciousness related to appearance and voice, and social confidence. Based on this review, a preliminary pool of items was generated and subsequently refined through informal expert consultation involving clinicians experienced in head and neck oncology and rehabilitation. To ensure clarity and face validity, the draft questionnaire was pilot-tested in a small group of patients with and without tracheostomy (not included in the final analysis). Minor linguistic adjustments were made to improve item comprehensibility. Formal test–retest reliability and factor analysis were not performed, which represents a limitation of the present study. However, internal consistency of the psychosocial barrier scale was high (Cronbach’s alpha = 0.89), supporting the coherence of the construct measured. The instrument was designed for comparative group analysis rather than diagnostic purposes.

All items were phrased to reflect a potential psychosocial barrier or negative perception, such that higher scores indicated greater psychosocial burden (e.g., stronger agreement signifying increased perceived stigma or social isolation). In addition to the core psychosocial items, the questionnaire gathered information on support systems and coping strategies, including participation in patient support groups and use of psychological counseling. The control group completed a nearly identical version of the instrument, with minor wording adaptations applied to ensure semantic equivalence of items across groups to reflect the absence of a tracheostomy (e.g., items referring to voice changes or surgical scars rather than the tracheostoma itself). In the control group, items referring explicitly to the tracheostomy were rephrased to refer to illness-related voice or appearance changes, preserving semantic equivalence. No items were added or removed, and the same composite score structure was applied in both groups. The questionnaire underwent pilot testing on a small group of patients (not included in the final analysis) to confirm clarity, face validity, and item relevance. Internal consistency for the psychosocial barrier scale was high (Cronbach’s alpha = 0.89), indicating that the items measured a coherent underlying construct. Item-level distributions and missing data were minimal and did not materially affect the presented results; detailed distributions can be provided upon reasonable request. Quantitative indices of content validity (e.g., CVI) were not calculated.

The primary outcome of interest was the overall level of psychosocial barriers related to social interaction and perceived acceptance, operationalized through a composite *Psychosocial Barrier Score*. This aggregate score was computed by summing Likert-scale responses across the five core items (possible range: 5–25), with higher scores reflecting greater psychosocial difficulty. Individual item responses were additionally examined to identify specific areas of psychosocial vulnerability, such as public self-consciousness or avoidance of social situations. Secondary outcomes focused on subgroup differences according to gender (male vs. female) and age. For age-stratified analyses, participants were categorized into two groups, i.e., adults younger than 60 years and those aged 60 years or older approximating the median age distribution of the sample.

### 2.3. Statistical Analysis

All statistical analyses were performed using IBM SPSS Statistics, version 27 (IBM Corp., Armonk, NY, USA). Descriptive statistics were used to characterize the study sample in each group. Continuous variables (e.g., age) are presented as means with standard deviations (SD), while categorical variables (e.g., sex, tumor stage) are reported as frequencies and percentages. The primary psychosocial outcome was treated as a continuous variable. Accordingly, between-group differences were assessed using independent-sample *t*-tests. Logistic regression analyses were not performed, as the study was not designed to estimate adjusted odds ratios or to support causal inference. Between-group comparisons were conducted using statistical tests appropriate to variable type and distribution. Continuous variables were compared using independent-sample *t*-tests for normally distributed data and the Mann–Whitney U test for non-normally distributed data. Categorical variables were analyzed using Chi-square (χ^2^) tests. All χ^2^ analyses met the required assumptions, including minimum expected cell counts. For psychosocial outcomes, the primary analysis compared mean composite Psychosocial Barrier Scores between the tracheostomy and control groups using an independent-sample *t*-test. In addition, item-level questionnaire responses were examined. For interpretability, Likert-scale responses were dichotomized, with “agree” and “strongly agree” classified as endorsement of a psychosocial barrier and all remaining responses classified as non-endorsement. Group differences in endorsement rates for individual items were evaluated using χ^2^ tests. Exploratory subgroup analyses were conducted to examine potential gender- and age-related differences within each study group. Gender-based comparisons of mean Psychosocial Barrier Scores were performed using independent-sample *t*-tests, with item-level differences were assessed analogously. Age-related analyses were conducted by stratifying participants into two predefined categories: younger adults (<60 years) and older adults (≥60 years). Mean Psychosocial Barrier Scores were compared between age groups within each study arm using independent-sample *t*-tests. To explore potential interaction effects between age category and group status (tracheostomy vs. control), a two-way analysis of variance (ANOVA) was performed. All statistical tests were two-tailed, and a significance threshold of *p* < 0.05 was applied. Adjustment for multiple comparisons and formal moderation analyses were not conducted, as the study was exploratory in nature and not powered for complex interaction modeling. The analyses were designed to address group-level differences in psychosocial burden rather than to model tracheostomy status as a dependent variable. Accordingly, multivariable logistic regression and odds ratio estimation were not applied. These limitations are acknowledged as areas for future research.

## 3. Results

A total of 300 patients participated in the study, with 150 in the tracheostomy group and 150 in the control group. Table 1 summarizes the demographic and clinical characteristics of both groups. The two groups were well-matched in terms of age, sex, primary tumor site, and disease stage at diagnosis. The mean age in the tracheostomy group was 59.2 years (SD = 9.4, range 38–78) and in the control group was 60.1 years (SD = 9.1, range 40–80), a difference that was not statistically significant (*p* = 0.45). Each group contained 105 males (70%) and 45 females (30%). Cancer diagnoses were comparable: in both groups, the majority of patients (approximately 80%) had primary laryngeal carcinoma, with the remaining having tumors of the trachea or other adjacent airway sites. Disease stage distribution was also similar; about four-fifths of the patients in each group had advanced-stage (Stage III or IV) disease at diagnosis, reflecting the inclusion criteria that led many to require aggressive treatment. Because of the matching design, none of the baseline differences between the tracheostomy and control groups reached statistical significance (all *p* > 0.20), indicating that any differences observed in psychosocial outcomes are less likely to be due to confounding by these characteristics. Table 1 presents the mean and standard deviation of the composite Psychosocial Barrier Score in the tracheostomy and control groups, including stratification by age and gender.

### Group Differences in Psychosocial Barriers

Patients in the tracheostomy group reported substantially more psychosocial difficulties related to social interactions and acceptance than those in the control group. The mean Psychosocial Barrier Score (composite of the survey items) was significantly higher in tracheostomized patients (mean score = 21.4 ± 4.8 on a 5–25 scale) compared to the control patients (mean = 12.3 ± 3.7), with a mean difference of approximately 9 points (*p* < 0.001, 95% confidence interval of difference: 8.0 to 10.0). This indicates that, on average, patients with a tracheostomy experienced many more psychosocial barriers.

Differences were also evident when examining individual questionnaire items. Table 2 presents a comparison of selected key survey items between the two groups, showing the proportion of patients who agreed or strongly agreed with each statement (thus acknowledging that particular psychosocial barrier). Across all items, a markedly higher fraction of tracheostomy patients endorsed negative psychosocial statements compared to control patients. For example, 60% of the tracheostomy group agreed that they often avoid social gatherings because of their condition, whereas only 15% of the control group reported doing so (*p* < 0.001). Similarly, a large majority of those with tracheostomy (72%) felt that strangers tend to stare at them due to the tracheostomy or changes in their voice, in contrast to only 10% of control patients who felt conspicuous in public (*p* < 0.001). Around two-thirds (66%) of tracheostomized patients expressed worry that healthy people feel uncomfortable interacting with them (e.g., due to the breathing tube or altered voice), while this concern was reported by just 18% of controls (*p* < 0.001). Moreover, 55% of the tracheostomy group admitted to feeling less confident in social situations post-surgery, compared to 20% in the control group (*p* < 0.001). About half (49%) of tracheostomy patients believed that some people avoid them because of their appearance or speech, versus 12% of control patients (*p* < 0.001). The magnitude of the between-group difference in the composite Psychosocial Barrier Score was large, indicating a large absolute difference between groups. These results indicate a consistent pattern: the presence of a tracheostomy is associated with a greatly increased perception of social stigma or awkwardness, leading to avoidance and reduced social participation.

Overall, patients with tracheostomy consistently reported higher psychosocial burden across all assessed domains compared with controls, confirming a robust association between tracheostomy and perceived social stigma.

We observed notable differences in psychosocial outcomes when comparing male and female patients, particularly in the tracheostomy group. Female patients tended to report higher psychosocial barrier scores than male patients. In the tracheostomy group, the mean Psychosocial Barrier Score for females was 23.5 ± 4.2, significantly greater than the mean of 20.3 ± 4.7 for males (*p* = 0.003). This suggests that women with a tracheostomy experienced more severe psychosocial challenges on average than their male counterparts. By contrast, in the control group, female patients had a mean score of 12.8 ± 3.9 versus 11.9 ± 3.5 in males, a small difference that was not statistically significant (*p* = 0.28). Thus, the gender disparity in psychosocial burden was pronounced only in those who had a tracheostomy.

Table 3 presents a breakdown of psychosocial scores by gender within each group. It also includes a comparison of the percentage of patients agreeing to certain key psychosocial items by gender. For instance, among tracheostomized patients, 80% of women vs. 55% of men agreed with the statement “I feel people stare at me,” and 60% of women vs. 45% of men reported feeling socially unconfident after the surgery. These differences (women higher than men) were significant. In the control group, there were minimal gender differences in item responses (for example, women and men had similarly low rates of agreement with such statements, around 15% or less, with *p*-values > 0.1 for gender difference in each case).

These findings illustrate that the psychosocial impact of a tracheostomy may be associated with differences by gender and age, with female patients in our study showing greater vulnerability to issues like social stigma and self-image disturbances. Male patients with tracheostomy, while still affected, tended to have slightly lower barrier scores and were less likely to endorse some of the appearance-related or social anxiety statements. Age also appeared to influence psychosocial outcomes to some degree. We stratified the sample at 60 years of age to compare younger vs. older patients. Among tracheostomy patients, those under 60 years old had a mean Psychosocial Barrier Score of 22.7 ± 4.6, compared to 20.1 ± 4.7 in those aged 60 and above (*p* = 0.01), indicating that younger patients with a tracheostomy were experiencing more psychosocial difficulty on average than the older ones. In contrast, for control patients without a tracheostomy, the mean score was 13.0 ± 3.8 in the younger subgroup and 11.8 ± 3.5 in the older subgroup, a difference that was not statistically significant (*p* = 0.08). Table 4 shows the comparison by age category. It also highlights that younger tracheostomy patients were more likely to agree with statements reflecting social anxiety or stigma. For example, 70% of younger tracheostomy patients (age < 60) agreed they avoid social gatherings, versus 50% of the older tracheostomy patients (*p* = 0.04 for the age difference within the tracheostomy group). No such age-related pattern was seen in the control group’s responses, which remained low in both age subsets.

## 4. Discussion

This study adds novel evidence by isolating the psychosocial impact of permanent tracheostomy through a matched comparison with non-tracheostomized patients, specifically focusing on perceived social stigma rather than general quality-of-life outcomes. The findings identify specific psychosocial barriers (such as perceived public scrutiny and social avoidance) that may guide the development of targeted psychosocial and rehabilitative interventions. Results indicate that the negative psychosocial effects associated with having a tracheostomy are more pronounced in younger patients (who may have higher social expectations or feel more disruption in work and family life) and in female patients (who may be more affected by changes in physical appearance and social roles). This case–control study examined psychosocial barriers (particularly perceptions of social acceptance) among patients with respiratory tract cancers, comparing those living with a tracheostomy to similar patients without a tracheostomy. The findings confirm that the presence of a tracheostomy is associated with significantly heightened psychosocial challenges. Patients with tracheostomies reported greater social avoidance, lower self-confidence in interactions, and an increased sense of being stigmatized or treated differently by others. In contrast, patients without tracheostomies (despite having had cancer) showed comparatively fewer social difficulties. These results align with the broader understanding that visible and functional alterations from cancer treatment can profoundly impact a patient’s psychosocial well-being [3,5]. For example, an observational study reported that quality of life was severely compromised in tracheostomized patients compared to those without tracheostomy up to one year after the procedure [3]. Likewise, head and neck cancer survivors often struggle with psychosocial adjustment when their treatment affects appearance or communication; in particular, a tracheostomy and related changes (like loss of natural voice) can lead to altered self-image and feelings of stigmatization [5,17]. Our findings echo these patterns: many tracheostomized patients felt “different” and less accepted, which manifested as withdrawal from social activities.

The psychosocial barriers observed (such as fear of being stared at or the belief that healthy individuals are uncomfortable around them) illustrate the concept of illness-related stigma. Patients with a visible stoma or breathing tube may internalize negative assumptions about how others perceive them [17]. This sense of stigma can be self-reinforcing, leading individuals to preemptively avoid social contact, thereby increasing isolation. Prior research supports the impact of perceived stigma in head and neck cancers, showing that stigma correlates with worse psychosocial outcomes [18]. Importantly, visible disfigurement due to cancer or its treatment has been identified as a key driver of cancer-related stigma [18]. In our study, virtually all tracheostomy patients had some visible alteration (the tracheostomy and often a neck scar), whereas the control patients (without a stoma) often had more subtle or internal treatment effects; this likely explains the large differences in perceived social acceptance between the groups. Interestingly, some literature has noted that reported stigma is sometimes lower than might be expected, suggesting variability in coping or societal attitudes. However, when present, stigma can still exert a powerful psychosocial impact [18]. Our data underscore that tracheostomy patients are a subgroup where stigma is especially salient—over half of them in our sample felt socially judged or avoided due to their condition.

Gender emerged as a significant factor in psychosocial outcomes post-tracheostomy. Female patients in the tracheostomy group had notably higher barrier scores and more frequent endorsement of feeling scrutinized or socially uncomfortable. This gender disparity is consistent with findings in other studies of laryngeal cancer survivors. For instance, women who undergo total laryngectomy (and thus have a permanent tracheostomy) have been reported to experience worse quality of life and social functioning than men [19]. One explanation is that societal expectations and self-image concerns may weigh more heavily on women; appearance changes (like a neck stoma) and voice alterations might interfere with roles and communication in ways that women find more distressing, given that women often place high importance on physical appearance and social connectedness [19,20]. Our female participants frequently mentioned issues like difficulty accepting the change in their neck appearance and concerns about femininity or attractiveness after surgery. They also tended to be more anxious about social rejection, which contributed to their higher barrier scores.

Not all studies, however, concur on gender effects; some research on cancer-related stigma has paradoxically found higher stigma scores reported by men (particularly in broader head and neck cancer populations) [18]. This may be related to factors such as risk-factor stigma (e.g., self-blame for tobacco use) or differences in reporting. It is important to note that the study showing higher stigma in men did not specifically focus on patients with visible disfigurement like tracheostomies. In contrast, in populations with an actual stoma or laryngectomy, women appear more adversely affected in psychosocial terms [19]. Thus, context is critical: women may be more vulnerable to the psychosocial impacts of disfigurement and voice loss, whereas men might experience other forms of stigma differently. Regardless, our findings highlight a need for heightened psychosocial support for female patients with tracheostomies [21]. Others have suggested that female laryngectomy patients could benefit from tailored counseling and support programs to address their unique concerns (such as body image and communication anxiety), and our results support this notion.

Age differences in our results indicate that younger patients struggled more with the psychosocial sequelae of tracheostomy than older patients. Younger adults (for example, in their 40s or 50s) often are in the midst of careers, active social lives, or raising families; an abrupt change like a permanent tracheostomy can significantly disrupt these activities and can be unexpected at that stage of life. They may feel a stronger sense of loss regarding normalcy and worry more about social and professional consequences [22,23]. Older patients, by contrast, might have a different baseline expectation of health in later life and often have established social circles that are more understanding or less centered on superficial appearance. Our finding that older tracheostomized patients had somewhat lower barrier scores suggests they had slightly better psychosocial adjustment. This trend parallels findings in the general oncology literature: older head and neck cancer patients sometimes report equal or even better quality of life in certain domains compared to younger patients [14]. Berg et al. observed that older patients (≥70 years) with head and neck cancer coped as well as or better than middle-aged patients in many quality of life aspects, except physical function [14]. Possible reasons include older adults having different coping strategies, a greater acceptance of health limitations, or less concern about social approval. In our study, older patients were less likely to perceive social rejection, which could reflect generational differences in self-consciousness or simply a smaller scope of social exposure (retirees versus working-age individuals) [24].

Despite these age and gender nuances, one common theme is clear: having a tracheostomy tends to amplify psychosocial challenges across the board. The device and its effects (e.g., altered speech, visible stoma and occasional mucus secretions or coughing) set patients apart in a visible way, which can hinder social reintegration after treatment. It is therefore imperative that cancer care teams address not just the medical management of patients with tracheostomies but also their psychosocial rehabilitation [21]. Our findings support the call for comprehensive survivorship care that includes psychological support and social reintegration strategies. Patients may benefit from counseling (to help adjust self-image and coping strategies) [21], speech therapy and voice rehabilitation (to improve communication confidence), and peer support groups where they can meet others with similar experiences. Indeed, our findings suggest that interventions such as teaching patients how to care for and discreetly cover their stoma, using speaking valves or voice prosthetics when possible, and engaging family members in communication training can mitigate some barriers [25]. Furthermore, education of the general public and of patient communities about tracheostomies might reduce instances of staring or discomfort, thereby improving social acceptance. These findings suggest potential areas for supportive interventions, which should be evaluated in future interventional studies.

It is also notable that many of our control patients, who did not have a tracheostomy, still reported some psychosocial concerns (albeit at much lower rates than the tracheostomy group). This reminds us that even without visible signs, head and neck cancer survivors can feel vulnerable—some control patients mentioned hoarseness or scars that made them self-conscious, and some had anxiety about health or mortality that affected their social life. Thus, psychosocial care should extend to all cancer survivors, with particular attention given to those with additional burdens like a tracheostomy.

This study has several strengths. It examined a relatively large sample (N = 300 in total), which to our knowledge is a relatively large single-center comparative study specifically focusing on tracheostomy-related psychosocial effects in cancer patients. The case–control design (comparing tracheostomy patients to a matched control group) allowed us to isolate the impact of the tracheostomy by ensuring both groups were similar in cancer type, stage, and treatment context. By developing a targeted questionnaire, we were able to directly address nuances of social acceptance and stigma which might not be fully captured by generic quality-of-life instruments. The questionnaire demonstrated high internal consistency and was grounded in patient experiences, enhancing its content validity. Another strength is the analysis of subgroups by gender and age, which adds depth to our understanding of how psychosocial effects may vary across different patient demographics. This stratified approach provides more actionable insights—for instance, highlighting that younger patients and women might need extra support.

However, certain limitations should be acknowledged. First, the study is cross-sectional in nature; we assessed patients at a single point in time after treatment. Consequently, we cannot infer causality or changes over time. It is possible that some patients adapt better as more time passes, or that psychosocial barriers might diminish (or worsen) with longer follow-up, but our design did not capture longitudinal dynamics. Second, while our questionnaire was tailored to the study’s objectives, it was not a previously validated instrument. We took care to pilot-test it and achieved a good reliability score, but it remains a study-specific tool. Using non-standardized measures can limit comparability with other studies. Third, there is a potential for response bias. Patients who felt particularly strongly about their social situation might have been more inclined to participate (though we attempted to recruit consecutive follow-up patients to mitigate self-selection). Moreover, self-reported data are subject to social desirability bias; for example, a patient might under-report feelings of stigma out of embarrassment, or might over-report avoidance behaviors to emphasize their struggles. We assured anonymity in responses to encourage honesty, but some bias may remain.

Another limitation is related to the matching criteria. We matched groups on broad factors (site and stage), but there could still be differences in treatment experiences that we did not account for. For instance, the control group included patients treated with chemoradiotherapy, which can have its own side effects that influence psychosocial health (like persistent hoarseness or swallowing difficulties). Although none of the control patients had a visible stoma, some did have noticeable voice changes or surgical scars; variability in these factors could introduce heterogeneity in the control outcomes. We did not quantify voice quality or disfigurement objectively in this study—variables that might have been useful to measure. Additionally, our study was conducted at a single specialized center, which may limit generalizability. Practices in surgical and rehabilitative care (and the surrounding social support structures) can vary between institutions and regions. Patients in our cohort had access to a multidisciplinary team including oncology social workers and speech therapists; in settings without such resources, psychosocial outcomes might differ (potentially worse if support is lacking).

Finally, while we looked at gender and age, we did not deeply explore other factors that might influence psychosocial outcomes, such as socio-economic status, marital status, or cultural background. These factors can modulate how individuals perceive illness and stigma. For example, being in a supportive marriage might buffer some of the social isolation, or certain cultural norms might affect willingness to go out in public with a medical device. Future studies could incorporate these variables for a more nuanced analysis.

## 5. Conclusions

This study demonstrates that oncology patients living with a tracheostomy experience substantially greater psychosocial barriers than patients treated without a tracheostomy. Increased social avoidance, perceived stigma, and reduced confidence were particularly evident among younger and female patients. These findings highlight that the psychosocial consequences of tracheostomy extend beyond physical rehabilitation and require systematic attention within survivorship care. Addressing these challenges is essential for improving long-term quality of life and social reintegration in this patient population.

## 6. Clinical Implications and Future Directions

The results underscore the need to integrate structured psychosocial screening and support into routine follow-up care for patients with tracheostomy. Multidisciplinary interventions involving psychological counseling, speech and communication rehabilitation, and peer support programs may help reduce perceived stigma and social withdrawal. Particular attention should be given to younger and female patients, who appear to be at higher risk of psychosocial distress. Future research should focus on longitudinal assessment of psychosocial adaptation following tracheostomy and on validating disease-specific instruments for measuring social stigma and acceptance. Interventional studies evaluating the effectiveness of targeted psychosocial and rehabilitative programs are also warranted.

## Figures and Tables

**Table 1:** Demographic and Clinical Characteristics of Participants.

Characteristic *	Tracheostomy Group (*n* = 150)	Control Group (*n* = 150)	*p*-Value
Age, years (mean ± SD)	59.2 ± 9.4	60.1 ± 9.1	0.45
Age range (years)	38–78	40–80	-
Sex	105 M (70%), 45 F (30%)	105 M (70%), 45 F (30%)	0.99
Primary tumor site—Larynx	122 (81.3%)	120 (80.0%)	0.75
Primary tumor site—Other *	28 (18.7%)	30 (20.0%)	0.75
Stage III–IV at diagnosis	121 (80.7%)	117 (78.0%)	0.58
Stage I–II at diagnosis	29 (19.3%)	33 (22.0%)	0.58
Time since treatment (months)	12.5 ± 6.8	13.1 ± 7.0	0.56

* Other primary sites included tracheal tumors and subglottic or oropharyngeal tumors requiring airway management.

**Table 2:** Comparison of Psychosocial Barrier Survey Items Between Groups.

Items *	Tracheostomy Group (%)	Control Group (%)	*p*-Value
“I avoid social gatherings because of my condition.”	60% (90/150)	15% (23/150)	<0.001
“People stare at me because of the way I breathe or speak.”	72% (108/150)	10% (15/150)	<0.001
“I worry others are uncomfortable talking with me.”	66% (99/150)	18% (27/150)	<0.001
“I feel less confident socially now than before.”	55% (83/150)	20% (30/150)	<0.001
“Some people avoid me due to my appearance or voice.”	49% (74/150)	12% (18/150)	<0.001

* For each statement, percentages reflect those who answered “Agree” or “Strongly agree.” The number in parentheses indicates the count of patients out of 150 in that category.

**Table 3:** Psychosocial Barrier Scores by Gender in Each Group.

Group	Female	Male	*p*-Value *
Tracheostomy Group (*n* = 150)	23.5 ± 4.2 (*n* = 45)	20.3 ± 4.7 (*n* = 105)	0.003
Control Group (*n* = 150)	12.8 ± 3.9 (*n* = 45)	11.9 ± 3.5 (*n* = 105)	0.28

* Psychosocial Barrier Score range 5–25, higher = worse. Mean differences are comparisons using two-sample *t*-tests.

**Table 4:** Psychosocial Barrier Scores by Age Category in Each Group.

Group	<60 Years	≥60 Years	*p*-Value
Tracheostomy Group (*n* = 150)	22.7 ± 4.6 (*n* = 75)	20.1 ± 4.7 (*n* = 75)	0.010
Control Group (*n* = 150)	13.0 ± 3.8 (*n* = 75)	11.8 ± 3.5 (*n* = 75)	0.080

## Data Availability

The data presented in this study are available on request from the corresponding author due to privacy and ethical restrictions.

## Abbreviations

The following abbreviations are used in this manuscript:

CBT	Cognitive Behavioral Therapy
HNCPDS	Head and Neck Cancer Psychosocial Distress Scale
